# 1024. Using DOOR-MAT to Theoretically Compare Three Rapid Diagnostic Tests for Gram-Negative Bloodstream Infections in Immunocompromised Patients

**DOI:** 10.1093/ofid/ofab466.1218

**Published:** 2021-12-04

**Authors:** Lauren Groft, Mandee Noval, James Mease, J Kristie Johnson, Kimberly C Claeys

**Affiliations:** 1 The Johns Hopkins Hospital, Baltimore, MD; 2 University of Maryland Medical Center, Baltimore, Maryland; 3 University of Maryland School of Pharmacy, Baltimore, Maryland; 4 University of Maryland, Baltimore, MD

## Abstract

**Background:**

Molecular rapid diagnostic tests (RDTs) for bloodstream infections (BSI) utilize a variety of technologies and differ substantially in organisms and resistance mechanisms detected. RDT platforms decrease time to optimal antibiotics; however, data on RDTs in special populations, such as immunocompromised are extremely limited. This study aimed to compare theoretical changes in antibiotics based on differences in panel identification of organisms and resistance targets among three commercially available RDT panels.

**Methods:**

Retrospective cohort of immunocompromised patients treated for gram-negative BSI at University of Maryland Medical Center from January 2018 to September 2020. Immunocompromised was defined as active hematologic or solid tumor malignancy at time of BSI diagnosis, history of hematopoietic stem cell transplantation (HSCT) or solid organ transplantation (SOT), or absolute neutrophil count (ANC) < 1000 cells/mm^3^ at any time 30 days prior to BSI diagnosis. Verigene BC-GN was performed as standard of care. GenMark ePlex BCID and BioFire FilmArray BCID 2 results were assigned based on respective identifiable organism panels.

An infectious diseases clinician blinded to final antimicrobial susceptibility testing (AST) results used RDT results to assign antibiotic treatments for each platform. Decisions were referenced against *a priori* DOOR-MAT matrices. A partial credit scoring system (0 to 100) was applied to each decision based on final AST results. The mean and standard deviation (SD) were compared across panels using One-Way Repeated Measures ANOVA with modified Bonferroni for multiple comparisons.

**Results:**

A total of 146 patients met inclusion. Baseline characteristics are summarized in Table 1. The mean (SD) DOOR-MAT scores for the three RDT panels were: 86.1 (24.4) Verigene BC-GN vs. 88.5 (22.2) GenMark BCID vs. 87.2 (24.4) BioFire BCID 2. There was no statistically significant difference between the panels for DOOR-MAT score (*P*=0.6).

Table 1. Baseline Patient Characteristics and Organism Identification

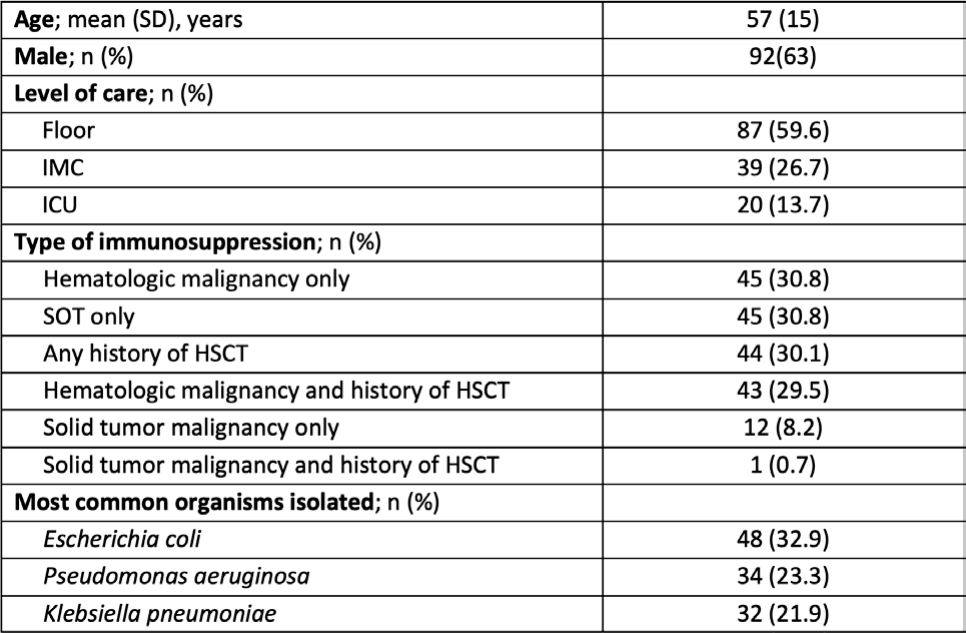

**Conclusion:**

Within an immunocompromised patient population, differences in organism identification between three commercially available RDT panels did not impact theoretical antibiotic prescribing.

**Disclosures:**

**J. Kristie Johnson, PhD, D(ABMM**), **GenMark** (Speaker’s Bureau) **Kimberly C. Claeys, PharmD**, **GenMark** (Speaker’s Bureau)

